# Self‐Gated Radial Free‐Breathing Liver MR Elastography: Assessment of Technical Performance in Children at 3 T

**DOI:** 10.1002/jmri.29541

**Published:** 2024-07-22

**Authors:** Sevgi Gokce Kafali, Bradley D. Bolster, Shu‐Fu Shih, Timoteo I. Delgado, Vibhas Deshpande, Xiaodong Zhong, Timothy R. Adamos, Shahnaz Ghahremani, Kara L. Calkins, Holden H. Wu

**Affiliations:** ^1^ Department of Radiological Sciences David Geffen School of Medicine, University of California Los Angeles Los Angeles California USA; ^2^ Department of Bioengineering University of California Los Angeles Los Angeles California USA; ^3^ US MR R&D Collaborations Siemens Medical Solutions USA, Inc. Salt Lake City Utah USA; ^4^ Physics and Biology in Medicine Interdepartmental Program, David Geffen School of Medicine University of California Los Angeles Los Angeles California USA; ^5^ US MR R&D Collaborations Siemens Medical Solutions USA, Inc. Austin Texas USA; ^6^ Department of Pediatrics, David Geffen School of Medicine University of California Los Angeles Los Angeles California USA

**Keywords:** free‐breathing, radial sampling, liver MR elastography, fibrosis, children, liver stiffness

## Abstract

**Background:**

Conventional liver magnetic resonance elastography (MRE) requires breath‐holding (BH) to avoid motion artifacts, which is challenging for children. While radial free‐breathing (FB)‐MRE is an alternative for quantifying liver stiffness (LS), previous methods had limitations of long scan times, acquiring two slices in 5 minutes, and not resolving motion during reconstruction.

**Purpose:**

To reduce FB‐MRE scan time to 4 minutes for four slices and to investigate the impact of self‐gated (SG) motion compensation on FB‐MRE LS quantification in terms of agreement, intrasession repeatability, and technical quality compared to conventional BH‐MRE.

**Study Type:**

Prospective.

**Population:**

Twenty‐six children without fibrosis (median age: 12.9 years, 15 females).

**Field Strength/Sequence:**

3 T; Cartesian gradient‐echo (GRE) BH‐MRE, research application radial GRE FB‐MRE.

**Assessment:**

Participants were scanned twice to measure repeatability, without moving the table or changing the participants' position. LS was measured in areas of the liver with numerical confidence ≥90%. Technical quality was examined using measurable liver area (%).

**Statistical Tests:**

Agreement of LS between BH‐MRE and FB‐MRE was evaluated using Bland–Altman analysis for SG acceptance rates of 40%, 60%, 80%, and 100%. LS repeatability was assessed using within‐subject coefficient of variation (wCV). The differences in LS and measurable liver area were examined using Kruskal–Wallis and Wilcoxon signed‐rank tests. *P* < 0.05 was considered significant.

**Results:**

FB‐MRE with 60% SG achieved the closest agreement with BH‐MRE (mean difference 0.00 kPa). The LS ranged from 1.70 to 1.83 kPa with no significant differences between BH‐MRE and FB‐MRE with varying SG rates (*P* = 0.52). All tested methods produced repeatable LS with wCV from 4.4% to 6.5%. The median measurable liver area was smaller for FB‐MRE (32%–45%) than that for BH‐MRE (91%–93%) (*P* < 0.05).

**Data Conclusion:**

FB‐MRE with 60% SG can quantify LS with close agreement and comparable repeatability with respect to BH‐MRE in children.

**Level of Evidence:**

2

**Technical Efficacy:**

Stage 1

Timely diagnosis and staging of liver fibrosis is needed for the management of a spectrum of liver diseases in adults and children.^1^, ^2^, ^3^, ^4^ Magnetic resonance elastography (MRE) can measure liver stiffness (LS), which is correlated with histopathological fibrosis staging.^2^, ^3^, ^4^ To avoid motion artifacts and measurement errors in the abdomen due to respiration, conventional liver MRE using Cartesian sampling requires breath‐holds (BH). In adults, the use of conventional BH Cartesian gradient‐echo (GRE) MRE has demonstrated promising results to detect and classify liver fibrosis with high sensitivity and specificity of 98% and 99%, respectively, when compared with liver biopsy.^4^ Recent improvements in BH‐MRE have enabled shorter scan times, better image quality, and lower technical failure rates. First of all, reduced repetition time (TR) achieved by rapid GRE wave encoding can cut the scan time in half and resulting images can still be used to quantify LS.^5^ Second, fractional encoding decreases echo time (TE), which can increase the signal‐to‐noise (SNR) ratio and leads to better image quality, especially in individuals that have shorter liver T_2_* (or longer R_2_*) due to iron overload.^6^ Previous work on rapid fractional Cartesian BH‐MRE reported larger measurable liver area on LS maps with respect to the numerical confidence masks when compared to BH‐MRE without rapid and fractional encoding.^7^ BH‐MRE also achieved lower technical failure rates than BH‐MRE without rapid and fractional encoding.^7^ As a result, Cartesian BH‐MRE is a technically reliable and clinically applicable method to quantify LS when BHs are feasible.

However, BHs are difficult in certain populations, such as those with decreased lung function, those who cannot follow instructions, obese people, elderly, and particularly children. Cartesian GRE BH‐MRE requires a series of consistent BHs (four or more) to acquire multiple slices in the liver. Inadequate or inconsistent BH can lead to poor image quality in children.^8^ Due to motion and other challenges, the sensitivity and specificity reported for Cartesian GRE BH‐MRE in children are lower than those reported for adults and can range from 33% to 88% and 89% to 94%, respectively.^1^, ^9^ To overcome this challenge, a child‐appropriate free‐breathing (FB) MRE technique that can accurately and rapidly quantify LS with minimal breathing motion artifacts is needed.

A few studies investigated Cartesian GRE FB‐MRE and reported varying degrees of agreement with respect to Cartesian GRE BH‐MRE in adults and children.^10^, ^11^, ^12^ On the other hand, non‐Cartesian radial trajectories are inherently more robust to motion and enable self‐navigated motion compensation.^13^ In children, FB radial MRI has achieved accurate and repeatable measurements for liver proton‐density fat fraction (PDFF) and R_2_* quantification.^13^ Based on these potential advantages, a recent study proposed radial GRE FB‐MRE, which achieved close agreement and comparable repeatability for measuring LS when compared to conventional Cartesian GRE BH‐MRE in 21 children at 3 T.^14^ While the initial results were promising, there were limitations. This previous radial FB‐MRE method required a relatively long scan time (2.5 minutes/slice) and coverage was limited to two slices due to the scan time. In addition, this previous radial FB‐MRE method used all of the data acquired during FB for image reconstruction and LS quantification. Although the radial sampling trajectory has inherent robustness to motion, using all of the FB data combines information from different respiratory motion states and may lead to suboptimal performance especially in individuals with irregular breathing patterns.^14^

In this study, our objectives were to develop an improved radial GRE FB‐MRE method with reduced scan time, increased liver coverage, and self‐gated (SG) motion compensation, and then assess its technical performance in children at 3 T.

## Materials and Methods

### Study Design

This Health Insurance Portability and Accountability Act compliant prospective study was approved by the Institutional Review Board. All parents/guardians provided written informed permission for their child to participate, prior to research procedures. Children provided assent when applicable. Inclusion criteria were 6–17 years of age and the ability to perform BH (duration around 15 seconds). Children older than 6 years of age were enrolled since most children can perform short BHs at this age. Exclusion criteria included congenital malformations of the liver, inborn error of metabolism, pregnancy, and contraindications to MRI such as claustrophobia and metallic objects in the body. All participants were instructed to fast and minimize fluid intake for 3–4 hours prior to the research MRI/MRE scan. For children undergoing a clinically indicated abdominal MRI examination, a 30‐minute research MRI/MRE examination was added to the clinical MRI examination. Children who did not have a clinically indicated abdominal MRI examination underwent a 90‐minute research‐only MRI/MRE examination. Data were entered into a secure database for management and analysis.^15^

### Research MRI/MRE Experiments

Participants were scanned using a research liver MRI/MRE protocol on a 3 T scanner (MAGNETOM PrismaFit, Siemens Healthineers, Erlangen, Germany) with an MRE system (Resoundant, Rochester, MN, USA) using 18‐channel body and 32‐channel spine array coils. The protocol included a product BH T_2_‐weighted half‐Fourier acquisition single‐shot turbo spin echo imaging (HASTE) sequence for anatomical reference, a product three‐dimensional (3D) Cartesian BH Dixon sequence with six TEs for liver PDFF and R_2_* quantification, and a product Cartesian GRE BH‐MRE sequence and a research application radial GRE FB‐MRE sequence for LS quantification.^16^, ^17^ The key imaging parameters for BH T_2_‐weighted HASTE and 3D Cartesian BH Dixon are summarized in Table S1 in the Supplemental Material.

To improve participants' comfort, a prototype flexible MRE passive driver (Mayo Clinic, Rochester, MN, USA) was used. The mechanical wave amplitude ranged from 30% to 60%, and was set depending on the discretion of the MR technologist and body mass index (BMI) of the participant according to the usage guidelines of the flexible MRE passive driver.^18^ The mechanical wave amplitude was matched for BH‐MRE and FB‐MRE techniques in each participant.

### MRE Image Acquisition

**FIGURE 1:**
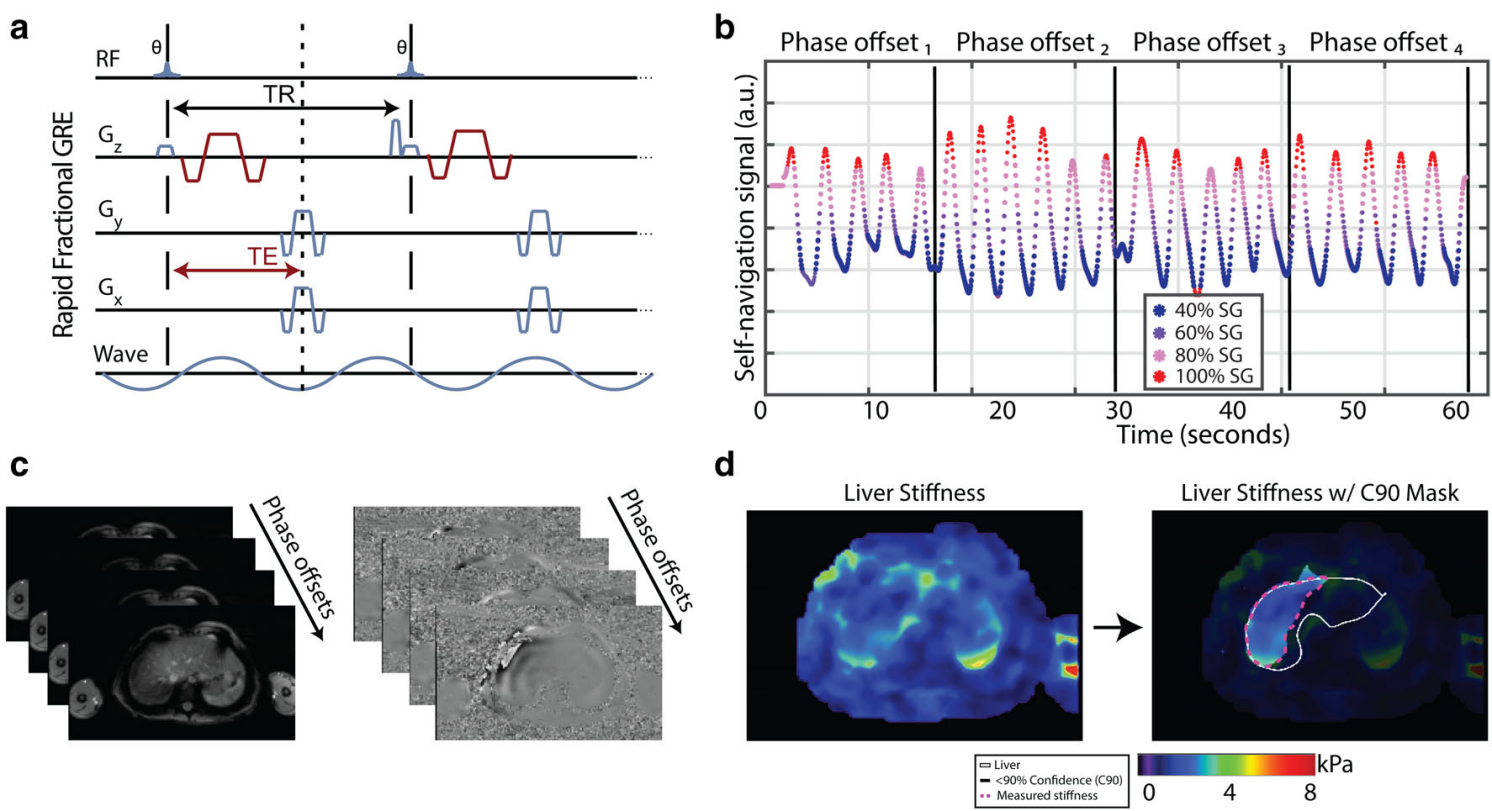
Radial rapid fractional free‐breathing (FB)‐MRE data acquisition, self‐gating, and processing. (**a**) Sequence diagram for rapid fractional GRE‐based radial FB‐MRE. (**b**) The center of k‐space self‐navigation signal that characterizes the breathing motion for a representative participant. The different colors indicate data selected with different self‐gating (SG) acceptance rates for image reconstruction. (**c**) Reconstructed magnitude and phase images for radial FB‐MRE with 60% SG at four wave phase offsets. (**d**) Using the stiffness maps from scanner software, liver stiffness was measured in regions of interest (magenta contour) in the liver (white contours) with ≥90% numerical confidence (regions not in the black masks).

A previous version of the radial FB‐MRE method used golden‐angle ordering, acquired two slices in 5 minutes and 26 seconds with nominally fully sampled k‐space according to Nyquist criteria, and did not explicitly perform motion compensation.^14^ This study developed an improved version of radial FB‐MRE by implementing additional features such as gradient delay corrections and rapid wave and fractional encoding.^5^, ^6^, ^19^, ^20^ Before the imaging data were acquired, gradient calibration lines for k‐space data along *k*_*x*_ and *k*_*y*_ directions were acquired. The k‐space shifts induced by the gradient delays were then calculated using cross correlation of the *k*_*x*_ and *k*_*y*_ calibration lines and corrected.^19^, ^21^ The sequence diagram for radial FB‐MRE using rapid and fractional encoding is shown in Fig. 1a.^5^, ^6^ Rapid wave encoding allows accurate measurements of LS with shortened scan time; TR is synchronized with 1.5 cycles of external motion (CEV) instead of 3 CEV, thereby enabling shorter TR. Additionally, the polarities of motion encoding gradients (MEGs) stayed the same for all TR cycles to generate the required phase contrast MR images with appropriate synchronization of MEGs to CEV.^5^

On top of rapid wave encoding, all MRE sequences in this study utilized fractional encoding, which decreases TE by reducing the encoding duration of the MEGs.^6^ Although the degree of wave encoding is decreased, previous studies have shown that fractional encoding achieves accurate measurement of LS.^7^ Imaging parameters such as TR, TE, flip angle, acquired and reconstructed in‐plane resolution, and the degree of fractional encoding were matched as much as possible for BH‐MRE and FB‐MRE (Table 1). All scans were acquired in the axial orientation.

**Table 1:** Representative Imaging Parameters

	Cartesian Rapid Fractional BH‐MRE	Radial Rapid Fractional FB‐MRE
TE	15.49 msec	16.10 msec
TR	25 msec	25 msec
Flip angle	12 degrees	12 degrees
Field of view (FOV)	350 × 285 mm^2^	350 × 350 mm^2^
Acquired matrix size	128 × 104	128 × 128
Recon. matrix size	256 × 208	256 × 256
Recon. pixel size	1.4 × 1.4 mm^2^	1.4 × 1.4 mm^2^
Acquired resolution	2.8 × 2.8 mm^2^	2.8 × 2.8 mm^2^
Interpolation	On	On
Radial views	NA	302
Oversampling factor	NA	1.5
Self‐gating acceptance rate	NA	40%, 60%, 80%, 100%
PAT (GRAPPA) factor	2	Off
Fractional encoding	65%	65%
Wave amplitude	30%–70%	30%–70%
Slice thickness	5 mm	5 mm
Scan time per slice	11 seconds (1 BH)	1 minutes 2 seconds (FB)^a^
Number of slices	4	1
Total scan time	1 minutes 29 seconds^b^ (4 BH, 4 slices)	4 minutes 8 seconds (4 slices)

The parameters for the rapid fractional Cartesian breath‐holding (BH)‐MRE and radial free‐breathing (FB)‐MRE sequences were matched as much as possible. All scans were acquired in the axial orientation at 3 T.

TE = echo time; TR = repetition time; BW = readout bandwidth; MEG = motion encoding gradients; PAT = parallel imaging; GRAPPA = generalized auto‐calibrating partial parallel acquisition; NA = not applicable.

^a^ This includes 1.5× oversampling of radial k‐space with respect to the Nyquist criteria.

^b^ This includes the BH instruction times, BH scan times, and minimum time breaks in between breath‐holds. The actual scan time might be longer.

To assess the intrasession repeatability, BH‐MRE and FB‐MRE were each repeated twice in the same examination without repositioning of the participants or moving the table.^22^ The first scan (scan 1) had a set order of BH‐MRE followed by FB‐MRE, and the order of the sequences was randomized for the second scan (scan 2).

### MRE Image Reconstruction

**FIGURE 2:**
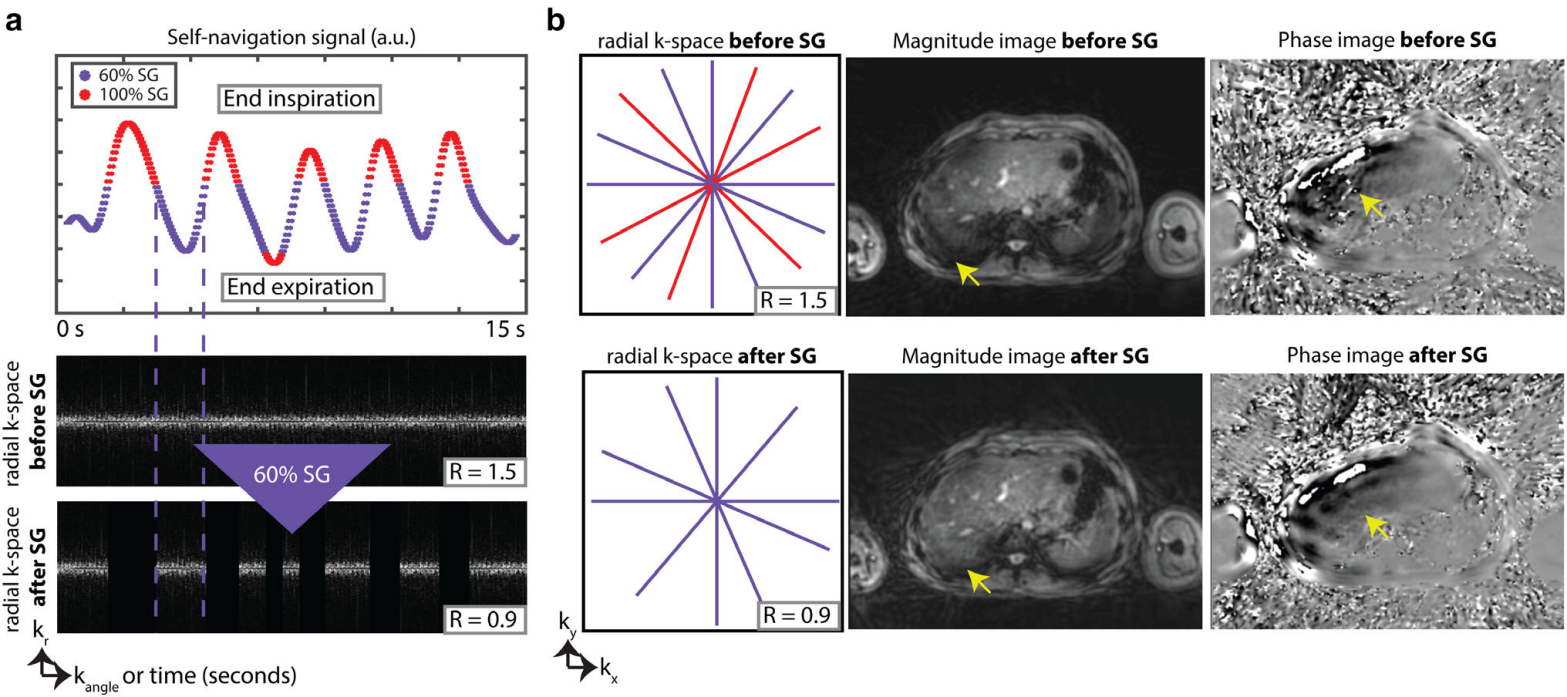
Details about self‐gated reconstruction for radial rapid fractional free‐breathing MRE. The examples shown here are from the second wave phase offset. (**a**) Representative self‐navigation curve and corresponding radial k‐space readouts (plotted in k_r_ and k_angle_) over time before and after self‐gating (SG) for a 16.3‐year‐old male with body mass index of 23.6 percentile. Example shown for 60% SG acceptance rate. As illustrated in the representative self‐navigation curve, the k‐space radial readouts (i.e., data points) closer to end‐expiration are selected during SG. Since the acquisition had 1.5× oversampling, the net sampling factor (R) after SG is 0.9 according to the Nyquist criteria. (**b**) Corresponding k‐space sampling trajectories in the *k*_*x*_–*k*_*y*_ plane for this wave phase offset, as well as reconstructed magnitude and phase images, before and after SG reconstruction. The signal dropouts in the liver before SG are recovered after SG reconstruction, as shown by yellow arrows.

After FB‐MRE data were acquired, SG image reconstruction was performed using a vendor provided framework.^13^, ^21^ K‐space center points of the radial readouts from each TR were sampled to obtain a self‐navigation signal time curve which characterizes the underlying breathing motion. According to a predetermined SG acceptance rate, the k‐space data points (i.e., radial readouts) that were closer to end‐expiration for the entire FB duration were selected for further processing and reconstruction.^21^, ^23^ Figure 2a shows a representative graph of k‐space radial readouts over time before/after SG and a representative self‐navigation curve using a 60% SG acceptance rate as an example. Representative k‐space sampling patterns in the *k*_*x*_–*k*_*y*_ plane with corresponding magnitude and phase images are shown for 60% and 100% SG in Fig. 2b.

The investigation of SG acceptance rates is important to analyze the trade‐off between motion state consistency versus the amount of data used for reconstruction, which affects the artifact and signal levels in the reconstructed images.^24^ For this purpose, this study tested SG acceptance rates of 40%, 60%, 80%, and 100%. Note that 100% SG indicates no data rejection, as all the radial readouts during FB were used for reconstruction. To reduce undersampling artifacts after SG motion compensation, FB‐MRE was prospectively oversampled by a factor of 1.5× (based on Nyquist criteria), resulting in net sampling factors of *R* = 1.2, 0.9, and 0.6 for SG acceptance rates of 80%, 60% and 40%, respectively.

All BH‐MRE and FB‐MRE images and LS maps were reconstructed offline using the scanner software provided by the vendor.

### LS Measurement

To avoid variability that might stem from region‐of‐interest (ROI) placement in the liver, this study used a semi‐automated way to measure LS. LS was measured inside ROIs in the liver with ≥90% numerical confidence (Fig. 1d).^1^, ^14^ First, the liver was contoured in each participant and slice by a researcher (S.G.K., with 5 years of experience analyzing MRE data) using the MRE magnitude images, and by considering BH T_2_‐weighted HASTE images as an anatomical reference. As per the guidelines from the Radiological Society of North America (RSNA) Quantitative Imaging Biomarkers Alliance (QIBA) Profile for Magnetic Resonance Elastography of the Liver, the portal vein was excluded while predominantly contouring the right lobe of the liver.^25^ By using a custom‐built in‐house software tool in MATLAB (Mathworks, Natick, MA, USA), an intersection mask between the liver contour and ≥90% numerical confidence (C90) mask was created automatically. This study adopted the threshold of ≥90% numerical confidence since it was previously used for LS measurement in children.^1^ LS for each slice was then calculated inside this mask, similar to a previous study.^26^ Outlier pixels with stiffness values that were smaller or greater than mean ± 1.5× IQR were removed, where IQR stands for the interquartile range.^27^ Finally, the measured LS was averaged across 4 slices, weighted according to the area of the final mask of each slice.

### Analysis of SG FB‐MRE Image Quality

**FIGURE 3:**
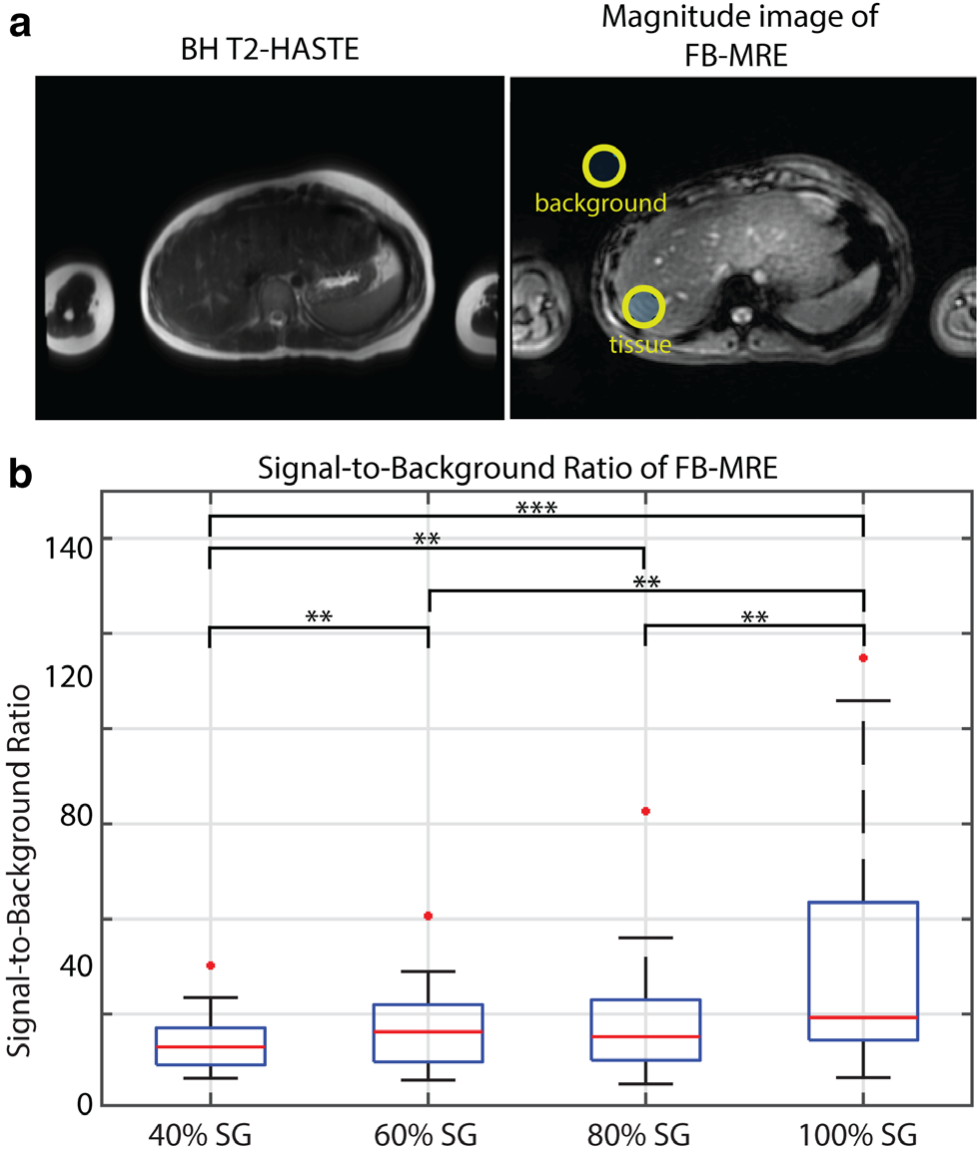
Signal‐to‐background ratio (SBR) of rapid fractional radial free‐breathing (FB)‐MRE with different self‐gating (SG) acceptance rates. (**a**) Example images showing the placement of the regions of interest in the liver and the background for measuring the SBR. A breath‐holding (BH) T_2_‐weighted HASTE image was used as an anatomical reference and the regions of interest were delineated on the corresponding FB‐MRE magnitude images from each wave phase offset. (**b**) Box‐whisker plots showing the distribution of SBR as a function of SG acceptance rate in 26 children. The SBR, which reflects the apparent signal‐to‐noise ratio (aSNR) and relative artifact level, varies across SG acceptance rates. SBR increases with less aggressive (i.e., higher) SG acceptance rates. ** and *** indicate *P* < 0.01 and *P* < 0.001, respectively.

The image quality of the FB‐MRE technique using different SG acceptance rates was assessed using the signal‐to‐background ratio (SBR). The SBR defined in this study characterized the combined effects of the apparent signal‐to‐noise ratio (aSNR) and the artifact level.^28^ The SBR was calculated as

(1) $$\mathrm{SBR}=\frac{\mu_{\text{tissue}}}{\sqrt{\frac{2}{4-\pi }\;}SD_{\mathrm{air}}}$$

where $\mu_{\text{tissue}}$ is the mean pixel intensity in the ROI at the target tissue and $SD_{\mathrm{air}}$ is the standard deviation of the pixel intensity in the background (i.e., air).^28^, ^29^ The ROIs were 5 cm^2^ in size and were placed by the same researcher who determined the liver ROIs on the MRE magnitude images, with the BH T_2_‐weighted HASTE images as an anatomical reference (Fig. 3a). SBR was measured in the first wave phase‐offset magnitude image of each slice for all participants, then averaged across slices to yield one SBR value for each participant.

### Analysis to Compare BH‐MRE and FB‐MRE

To compare the LS measured by the BH‐MRE and the FB‐MRE techniques, a series of metrics for agreement, repeatability, and technical quality were calculated.

Agreement between the LS measured from the second scans of BH‐MRE and FB‐MRE with varying SG acceptance rates (from 40% to 100%) was assessed using Bland–Altman (BA) analysis in terms of mean difference (MD) and 95% limits of agreements (LoA).^30^

According to the RSNA QIBA profile for liver MRE, the within‐subject coefficient of variation (wCV) was used to evaluate intrasession repeatability.^25^ wCV is formulated as

(2) $$\mathrm{wCV}=\sqrt{\sum_{i=1}^{N}\left(\mathrm{wS}D_{i}^{2}/Y_{i}^{2}\right)/N\;}$$

where N is the total number of participants, $\mathrm{wS}D_{i}^{2}$ is the within‐subject standard deviation (SD) of the LS measurements of participant i, and $Y_{i}$ is the mean of the LS measurements from two repeated scans of participant i.^25^ BA analysis was performed as an additional way to examine repeatability.^30^

Technical quality was assessed in terms of the measurable liver area (%) on LS maps, using the following ratio:

(3) $$\;\frac{A_{\text{liver}}\cap \left(A_{\text{total}}\geq C90\right)}{A_{\text{total}}\geq C90}$$

where $A_{\text{liver}}$ is the total liver area in all 4 slices, and $A_{\text{total}}\geq C90$ is the total area with numerical confidence greater than 90% in all four slices.^14^, ^31^

### Statistical Analysis

The differences between SBR of FB‐MRE using varying SG acceptance rates were examined. The differences in LS and the measurable liver area measured by BH‐MRE and FB‐MRE with varying SG acceptance rates were also analyzed.

For these purposes, the Kruskal‐Wallis test was used to analyze the groupwise differences. *P*‐values smaller than 0.05 were considered significant. Then, if significant differences were found, pairwise differences were analyzed using Wilcoxon signed‐rank tests after adjusting the *P*‐values for multiple comparisons (ten pairs) using Bonferroni correction to control the false discovery rate (FDR) to a level of 0.05. All continuous variables were reported in terms of median and [25^th^ percentile, 75^th^ percentile].

## Results

A total of 26 children without liver fibrosis were enrolled in this study (median age: 12.9 [10.6 years, 15.6 years] years; 15 females, 11 males). The study cohort consisted of 12 children with normal BMI (<85^th^ percentile), and 14 overweight children with BMI ≥ 85th percentile. Participant characteristics are reported in Table 2. One participant had a clinically indicated MRI examination with additional 30 minutes of research MRI/MRE, while the remaining 25 participants underwent dedicated research‐only MRI/MRE examinations for this study. The minimum scan duration for BH‐MRE was 1 minute and 29 seconds for 4 slices, which included the BH instruction time, BH scan time, and minimum time breaks needed between the BHs. The actual BH‐MRE scan time might be longer. The FB‐MRE scan took 4 minutes and 8 seconds to acquire 4 slices, which already included the 1.5× radial oversampling.

**Table 2:** Demographic Information for Children in This Study

	Participant Characteristics (*N* = 26)
Sex	15 female, 11 male

	Median	25th %ile, 75th %ile	Range [min, max]
Age	12.9 years	10.6 years, 15.6 years	[9.0 years, 17.6 years]
Body mass index	93.6th %ile	80.0th %ile, 98.6th %ile	[17.1th %ile, 99.5th %ile]
Liver PDFF	2.4%	1.8%, 5.5%	[1.2%, 27.6%]
Liver R_2_*	39.3 seconds^−1^	35.3 seconds^−1^, 44.4 seconds^−1^	[27.2 seconds^−1^, 84.3 seconds^−1^]

%ile = percentile; PDFF = proton‐density fat fraction.

**FIGURE 4:**
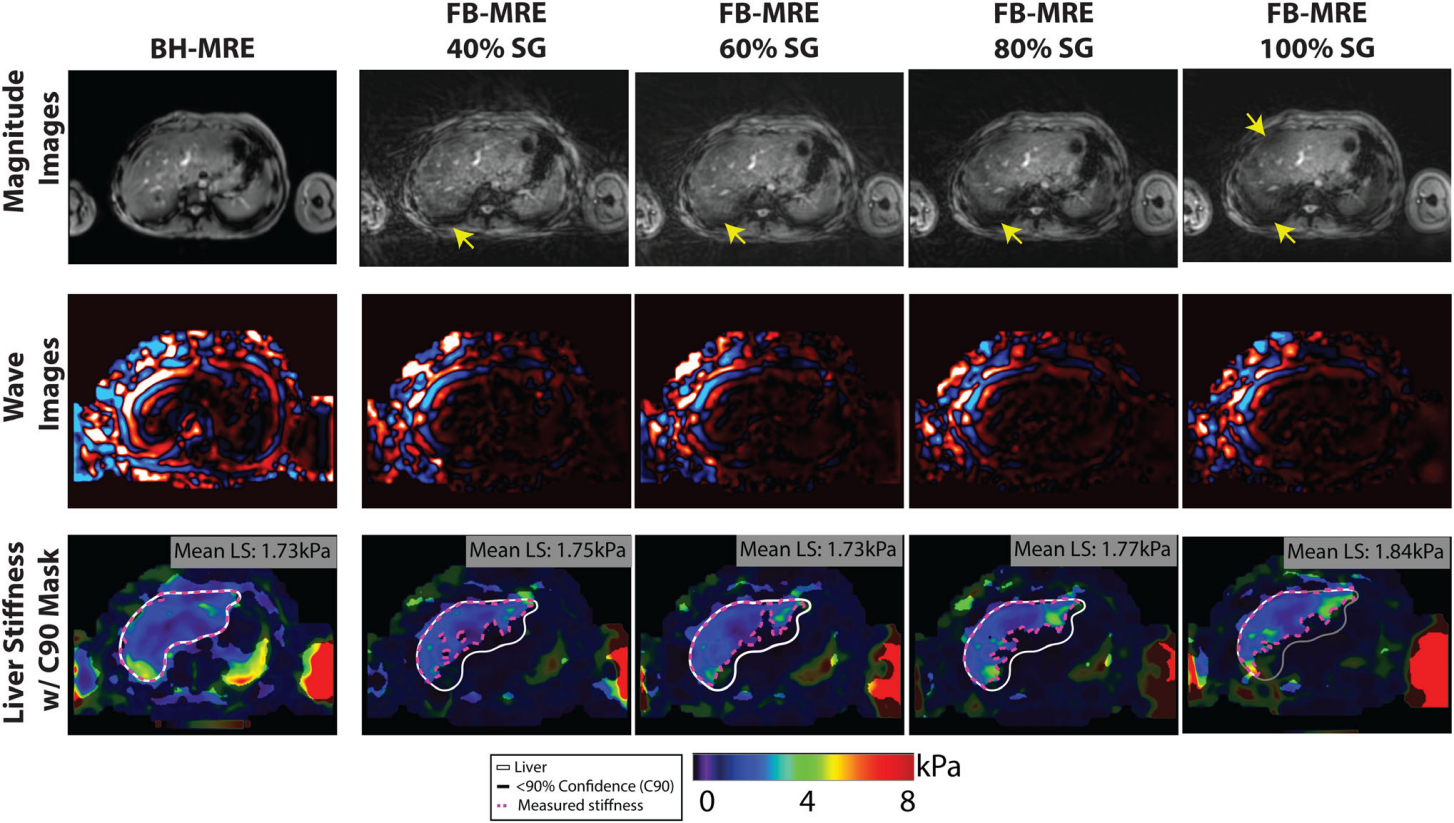
Representative axial magnetic resonance (MR) elastography images for rapid fractional Cartesian breath‐hold (BH)‐MRE and radial free‐breathing (FB)‐MRE sequences with varying self‐gating (SG) acceptance rates for a 16.3‐year‐old male with body mass index of 23.6 percentile. Magnitude images, wave images, and stiffness maps with ≥90% numerical confidence masks (C90) (darker regions indicate confidence <90%) and liver contours (white contours) are shown. Yellow arrows point out the motion‐induced signal dropouts in 100% and 80% SG, which are recovered by 60% and 40% SG. All tested MRE methods yielded consistent liver stiffness values ranging from 1.73 to 1.84 kPa, which was measured at the intersection between the liver contours and C90 masks (magenta contours).

**FIGURE 5:**
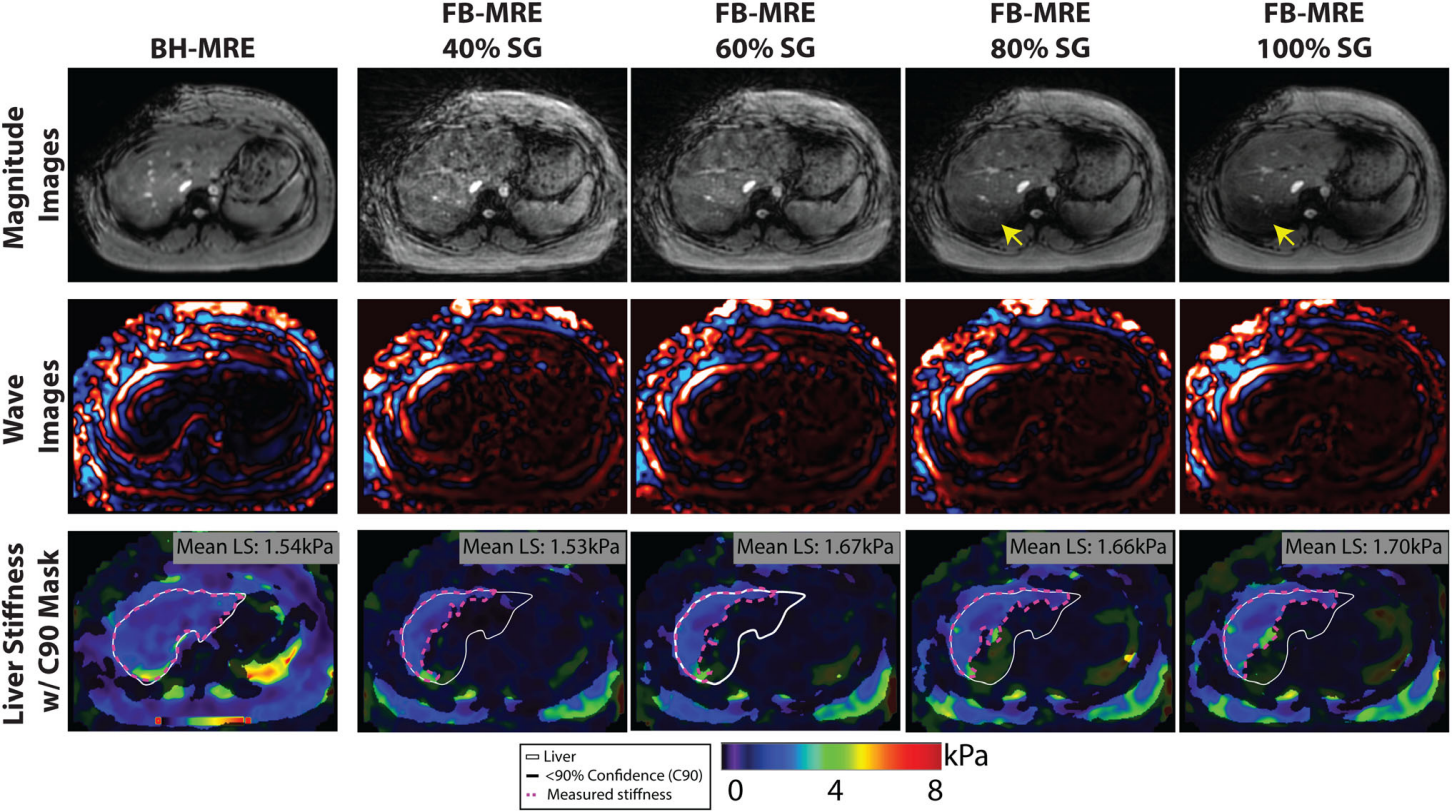
Representative axial magnetic resonance (MR) elastography images for rapid fractional Cartesian breath‐hold (BH)‐MRE and radial free‐breathing (FB)‐MRE sequences with varying self‐gating (SG) acceptance rates. This 15.9‐year‐old female participant has elevated body mass index of 96.7 percentile. Magnitude images, wave images, and stiffness maps with ≥90% numerical confidence masks (C90) (darker regions indicate confidence <90%) and liver contours (white contours) are shown. Yellow arrows point out the motion‐induced signal dropouts in 100% and 80% SG, which are recovered by 60% and 40% SG. All tested MRE methods yielded consistent liver stiffness values ranging from 1.54 to 1.70 kPa, which was measured at the intersection between the liver contours and C90 masks (magenta contours).

Figures 4 and 5 show example BH‐MRE and FB‐MRE results including magnitude images, wave images, and the corresponding LS maps overlayed on ≥C90 masks for two representative participants.

### Analysis of SG FB‐MRE Image Quality

The analysis of SBR for FB‐MRE with varying SG acceptance rates is summarized in Fig. 3b using box‐whisker plots. The highest median SBR of 25.02 (18.76, 56.72) was achieved without SG motion compensation (i.e., 100% SG acceptance rate) ($P_{\text{adjusted}}<0.05$) while the lowest median SBR of 16.97 (11.99, 22.12) were observed at 40% SG due to prominent streaking artifacts ($P_{\text{adjusted}}<0.05$). FB‐MRE with 80% and 60% SG achieved similar median SBR of 19.75 (13.28. 29.83) and 21.10 (11.83. 28.61), respectively ($P_{\text{adjusted}}=0.42$). Despite the high SBR, FB‐MRE with 100% SG led to signal dropouts in the right lobe of the liver due to motion in the head/foot direction whilst MRE at lower SG acceptance rates mitigated the signal dropouts by compensating for motion (Figs. 4 and 5).

### Analysis to Compare BH‐MRE and FB‐MRE

**FIGURE 6:**
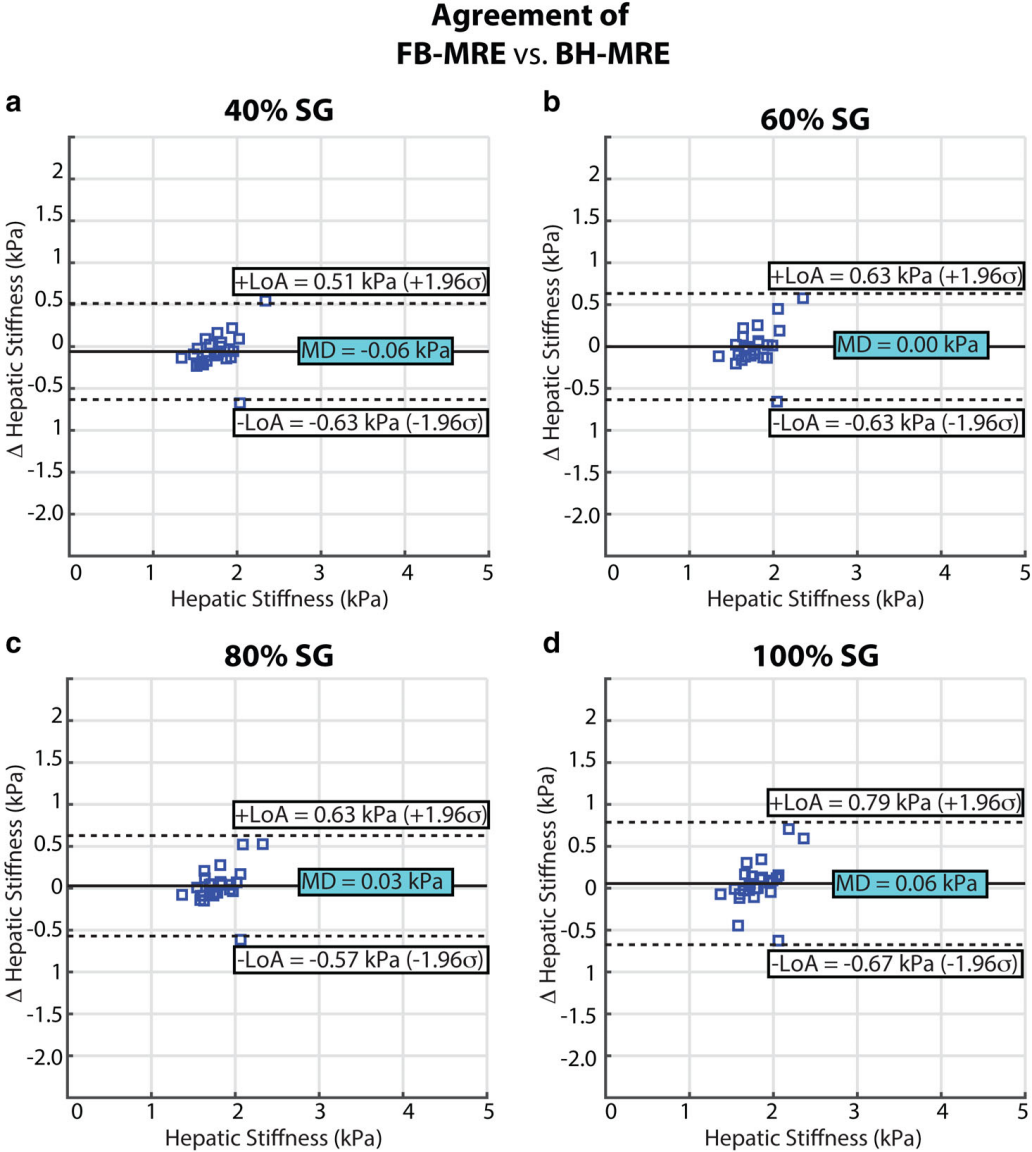
Agreement between liver stiffness (LS) values measured using rapid fractional Cartesian breath‐hold (BH)‐MRE and radial free‐breathing (FB)‐MRE with SG acceptance rates of (**a**) 40%, (**b**) 60%, (**c**) 80%, and (**d**) 100%. Bland–Altman analysis was performed in 26 children, and plots with mean difference (MD) and 95% limits of agreement (LoA) are shown. All radial FB‐MRE methods show close agreement of the LS measurements with respect to Cartesian BH‐MRE. Radial FB‐MRE with 60% SG acceptance rate yielded the smallest MD (0.00 kPa) with respect to Cartesian BH‐MRE when compared to radial FB‐MRE using other SG acceptance rates. σ = standard deviation of the difference in LS.

The BA plot in Fig. 6 shows the agreement of LS between BH‐MRE and FB‐MRE with varying SG acceptance rates. All FB‐MRE methods had small MD of LS (−0.06 to 0.06 kPa) with respect to BH‐MRE, and FB‐MRE with 60% had MD of 0.00 kPa compared with BH‐MRE (Figs. 4 and 6b). No differences in LS values were observed between BH‐MRE and FB‐MRE with varying SG acceptance rates in scan 1 and 2 (P=0.52).

**FIGURE 7:**
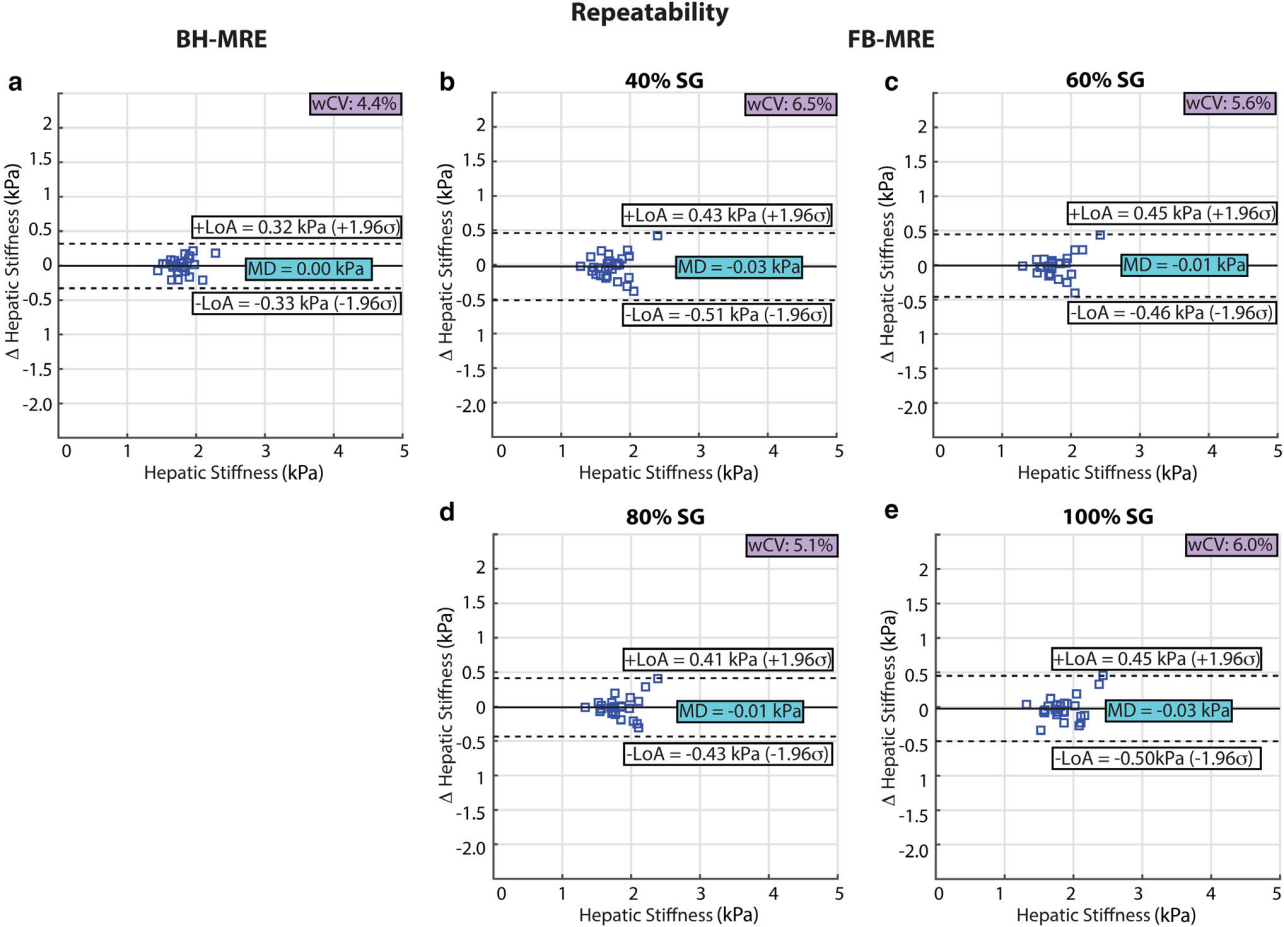
Repeatability of the liver stiffness (LS) values measured using (**a**) rapid fractional Cartesian breath‐hold (BH)‐MRE and radial free‐breathing (FB)‐MRE with SG acceptance rates of (**b**) 40%, (**c**) 60%, (**d**) 80%, and (**e**) 100%. Bland–Altman analysis was performed for repeated scans within the same session (26 children), and plots with mean difference (MD) and 95% limits of agreement (LoA) are shown. All of the evaluated MRE methods yielded repeatable LS measurements with MD close to 0 kPa. Cartesian BH‐MRE was the most repeatable method. The radial FB‐MRE method with 60% and 80% SG acceptance rates were more repeatable when compared to radial FB‐MRE with SG acceptance rates of 40% and 100% (no data excluded due to motion). σ = standard deviation of the difference in LS; wCV = within‐subject coefficient of variation.

Repeatability analysis with BA plots is illustrated in Fig. 7 for BH‐MRE and FB‐MRE with varying SG acceptance rates. All methods produced repeatable LS values with MD close to 0.00 kPa: BH‐MRE had MD of 0.00 kPa and LoA (0.65 kPa) followed by MD FB‐MRE using 60% and 80% SG acceptance rates (MD of −0.01 kPa for both). wCV was 4.4% for BH‐MRE. For FB‐MRE, wCV were 6.0% with 100% SG, 5.1% with 80% SG, 5.6% with 60% SG, and 6.5% with 40% SG.

Table 3 reports the measurable liver area in percentages for all tested MRE methods. Figure S1 in the Supplemental Material shows the box‐whisker plots for the measurable liver area. BH‐MRE achieved the greatest median measurable liver area of 93% and 91% for scan 1 and 2, respectively. Overall, FB‐MRE produced smaller median measurable liver area ranging from 32% to 45% across varying SG acceptance rates. Among all FB‐MRE sequences, 40% SG led to the smallest median measurable liver area for both scan 1 with median of 37% (27%, 53%) and scan 2 with median of 32% (27%, 47%). The largest measurable liver area for FB‐MRE was obtained when there was no SG motion compensation, i.e., 100% SG, for scan 1 with median of 45%, (33%, 58%) and scan 2 with median of 42% (34%, 56%). For scan 1, FB‐MRE with 60% and 80% SG acceptance rates produced median measurable liver area of 42% (35%, 57%) and 45% (37%, 55%), respectively. Scan 2 yielded median measurable area of 39% for FB‐MRE with SG acceptance rates of 60% (30%, 51%) and 80% SG (34%, 54%). The Kruskal‐Wallis test found significant differences between the MRE methods (*P* < 0.05). For scan 1 and 2, pairwise comparisons between the MRE methods (BH‐MRE, FB‐MRE with 40%, 60%, 80% and 100% SG) yielded significant differences for all pairs ($P_{\text{adjusted}}<0.05$) except for the pairs from FB‐MRE with 60% SG versus 80% SG acceptance rate ($P_{\text{adjusted}}=0.06$ for scan 1; $P_{\text{adjusted}}=0.05$ for scan 2), and 80% SG versus 100% SG acceptance rate ($P_{\text{adjusted}}=1.00$ for scan 1; $P_{\text{adjusted}}=0.09$ for scan 2).

**Table 3:** Measurable Liver Area in Percentages Reported as Median and (25th Percentile, 75th Percentile) for All Tested MRE Methods

Measurable Liver Area (%)		
MRE Method	Scan 1	Scan 2
Cartesian BH‐MRE	93%, (88%, 96%)	91%, (83%, 95%)
Radial FB‐MRE with 40% SG	37%, (27%, 53%)	32%, (27%, 47%)
Radial FB‐MRE with 60% SG	42%, (35%, 57%)	39%, (30%, 51%)
Radial FB‐MRE with 80% SG	45%, (37%, 55%)	39%, (34%, 54%)
Radial FB‐MRE with 100% SG	45%, (33%, 58%)	42%, (34%, 56%)

BH = breath‐hold; FB = free‐breathing; SG = self‐gating acceptance rate.

## Discussion

This study investigated the technical performance of an improved radial FB‐MRE method that was able to acquire 4 slices in ~4 minutes. When compared to no SG motion compensation, FB‐MRE using SG with 80% and 60% acceptance rates yielded closer agreement in LS with BH‐MRE. FB‐MRE with 60% SG, in particular, had closer agreement to BH‐MRE than the other SG acceptance rates. All tested MRE techniques achieved wCV <7%, indicating that a true change in LS of 19% can be detected with 95% confidence according to the QIBA profile for liver MRE.^25^ Although the FB techniques produced larger MD and wCV than those of the BH technique, all variations of FB‐MRE had consistent and acceptable repeatability according to the benchmark set by the QIBA profile for liver MRE. Among FB‐MRE methods, 60% and 80% SG produced the most repeatable LS values with the smallest wCV. 60% SG could be a good choice to balance quantitative accuracy and repeatability for LS measurement using radial FB‐MRE.

One of the ways to compensate for breathing motion during FB acquisition is to use retrospective SG reconstruction.^21^ This work analyzed the tradeoffs between SBR and motion compensation for FB‐MRE using different SG acceptance rates. SBR, which reflects apparent SNR and relative artifact level in magnitude images, increased with less aggressive (i.e., higher) SG acceptance rates, as more signal is averaged over the entire FB duration. When there was no data rejection based on the self‐navigation signal (i.e., 100% SG), SBR was the highest, leading to more measurable liver area. Yet, liver motion in the head/foot direction started to introduce motion inconsistencies in the data accepted for reconstruction, leading to the signal dropouts at the right lobe of the liver, and resulting in bias in LS measurements. This, in turn, reduced the agreement and repeatability of LS values with respect to BH‐MRE. With more aggressive (i.e., lower) SG acceptance rates and therefore smaller net sampling rates R, SBR decreased. 40% SG, which was the most aggressive SG acceptance rate tested in this study, suffered from radial undersampling artifacts, which reduced the agreement and repeatability in LS compared to BH‐MRE. For all these reasons, 60% SG, an intermediate level, may be a good option that addresses the trade‐off between SBR and motion compensation, while producing the closest agreement (smallest MD) and comparable repeatability vs. BH‐MRE. Future work can investigate constrained reconstruction to improve image quality for smaller net sampling rates that might stem from lower SG acceptance rates (i.e., 40%), or to avoid the need for oversampling of k‐space to save scan time. Note that the SG method used in this study only focused on extracting/accepting data at or near end‐expiration for reconstruction. With the same acquisition and more sophisticated SG algorithms, future work could potentially examine the LS across different motion states, similar to a previous study.^32^

The measurable liver area (in percentage) is one of the technical quality control metrics proposed for liver MRE.^14^, ^31^ While all variants of FB‐MRE in this work produced significantly smaller measurable liver area than BH‐MRE, the mean LS value from FB‐MRE achieved close agreement with that from BH‐MRE. This may be because the measurable liver areas on FB‐MRE included Couinaud segments such as IV, VIII, and V. Previous work has shown that the LS measured from these segments are highly correlated with the LS measurements in one global liver ROI.^33^ A study conducted in adults investigated potential factors influencing smaller measurable liver area for Cartesian GRE BH‐MRE and found that elevated liver PDFF and R_2_* might be contributing factors.^31^ For radial FB‐MRE, there could be some additional factors such as variable breathing patterns in children,^34^ which might contribute to smaller measurable liver area. Future work can investigate these specific factors, especially breathing patterns and elevated liver PDFF and R_2_*, in a larger population of children.

When compared to a previously proposed radial FB‐MRE method, the novelty of the FB‐MRE method presented in this study is 3‐fold.^14^ First, SG motion compensation was implemented and used to analyze the effects of varying SG acceptance rates on LS quantification of FB‐MRE with respect to reference BH‐MRE measurements. In children, SG motion compensation can be especially crucial due to variable breathing patterns, which can lead to more variable through‐plane displacement of the liver during axial scans.^34^ Second, the FB‐MRE method in our study reduced scan time by more than two‐fold compared to the previous method (1 minute/slice vs. 2.5 minutes/slice). The differences in scan time are based on a combination of improvements: the rapid wave encoding to cut TR and scan time in half and lower acquired in‐plane resolution to match conventional BH‐MRE. Both BH‐MRE and FB‐MRE acquired data with in‐plane resolution of 2.8 × 2.8 mm^2^, and then interpolated the images to yield 1.4 × 1.4 mm^2^ reconstructed in‐plane resolution to save scan time. Note that the reconstructed (interpolated) resolution (1.4 × 1.4 mm^2^) of BH‐MRE and FB‐MRE is used in clinical protocols at our institution, as well as in other previous work.^7^ These reductions in scan time allowed us to prospectively acquire FB‐MRE with 1.5× oversampling, which was beneficial for self‐gating, while maintaining acquisition time of 1 minute/slice. Third, due to reduced scan time per slice, FB‐MRE in this study could acquire four slices in the same total duration in which the previous radial FB‐MRE acquired two slices. As liver MRE typically acquires four mid‐liver axial slices, this improvement in the number of slices was highly relevant.

A few previous studies investigated FB‐MRE in adults and children using Cartesian readouts.^10^, ^11^, ^12^ Nevertheless, sequences with Cartesian trajectories are sensitive to artifacts caused by motion. Navigator triggering could be a potential remedy to this problem. Previous research in adults demonstrated a close agreement between navigator‐triggered Cartesian GRE FB‐MRE and Cartesian GRE BH‐MRE, with a concordance coefficient of 0.716.^15^ Another study investigated a FB‐MRE technique based on spin‐echo echoplanar imaging and reported the performance of prospective gating with navigator triggering in adults.^35^ Prospective gating works well when the breathing pattern is more stable or the liver displacement in the head‐foot direction is under certain limits. In adults, previous work reported accurate shear wave speed based LS measurements using prospective gating within an acquisition window defined by navigator signals.^35^ However, children frequently exhibit more variable breathing patterns, and navigator triggering may extend or introduce variability in scan durations while still exhibiting residual motion artifacts.^36^ Alternatively, a commercial respiratory bellows could be used to prospectively compensate for breathing motion with Cartesian acquisitions.^37^ When compared to Cartesian sampling, radial trajectories are more motion‐robust and can provide retrospective self‐gating for motion compensation, which allows fixed scan time without an external device like the bellows.^34^, ^38^ The relative benefits of prospective and retrospective gating for motion compensation in FB‐MRE could be analyzed in children as a future work.

This work used manual liver contours to measure LS, which could be further improved. Automated liver segmentation could help create an improved tool to measure LS. This way, clinicians and researchers might avoid user‐dependent errors that originate from manual liver contouring. Additionally, by replacing the lengthy manual liver contouring process with artificial intelligence‐based liver segmentation, this could speed up the LS measurements process. Future work could investigate artificial intelligence‐based automated liver segmentation for LS measurement.

The reference technique used in this study was BH‐MRE, which requires four separate and consecutive BHs. Thus, our inclusion criteria considered children who are already able to perform short BHs. Yet many children, such as young children, children with chronic diseases, and obese children, have a harder time performing BHs.^38^ Consequently, BH might not be as subject‐friendly or reliable in children as in adults. Once tested in children with fibrotic livers and in larger populations, FB‐MRE with SG could be used in children who have difficulty performing BHs.

### Limitations

Our study was a single‐site study with a small sample size. Moreover, this study did not evaluate the performance of FB‐MRE in diagnosing liver fibrosis, since it aimed to investigate the technical performance of FB‐MRE. Future works on a larger scale are warranted to assess the quantification accuracy as well as correlations with other quantitative markers such as PDFF and R_2_*. Second, the range of liver R_2_*, which is a biomarker for iron deposition, was still limited in our study, and future work could analyze the benefits of fractional encoding of FB‐MRE in a target population that has a wider range of liver R_2_* values. Third, similar to BH‐MRE, FB‐MRE currently acquires a single slice at a time. Future work might implement radial simultaneous multi‐slice imaging or incorporate 3D trajectories, such as TURBINE‐MRE, to further reduce the scan time for acquiring multiple slices.^39^, ^40^ Lastly, our radial FB‐MRE method is still a research application and is not yet available for widespread evaluation.

### Conclusion

This study demonstrated that the proposed rapid fractional radial FB‐MRE technique using a 60% SG acceptance rate effectively obtained four mid‐liver slices in 4 minutes. The measured LS value with this FB‐MRE technique showed close agreement and comparable repeatability with respect to the reference rapid fractional Cartesian BH‐MRE method. SG radial FB‐MRE can be a useful alternative in patients who have difficulty holding their breaths.

## Supporting information


**Data S1** Supporting Information.
