# Supplementary material for: Self‐Gated Radial Free‐Breathing Liver MR Elastography: Assessment of Technical Performance in Children at 3 T
Source: J Magn Reson Imaging. 2024 Jul 22;61(3):1271–83. doi: 10.1002/jmri.29541 (PMC11751131; doi:10.1002/jmri.29541)
Supplement: Supplementary file 1 — Data S1 Supporting Information. [file JMRI-61-1271-s001.docx]

**Supplementary Information for**

**“Self-Gated Radial Free-Breathing Liver MR Elastography:**

**Assessment of Technical Performance in Children at 3T”**

**Submitted to Journal of Magnetic Resonance Imaging**

Sevgi Gokce Kafali, M.S.,^1,2^ Bradley D. Bolster Jr., Ph.D.,^3^ Shu-Fu Shih, Ph.D.,^1,2^ Timoteo I. Delgado, B.A.,^1,4^ Vibhas Deshpande, Ph.D.,^5^ Xiaodong Zhong, Ph.D.,^1,2,4^ Timothy R. Adamos, M.D.,^6^ Shahnaz Ghahremani, M.D.,^1^ Kara L. Calkins, M.D., M.S.,^6^ and Holden H. Wu, Ph.D.^1,2,4^

^1^ Department of Radiological Sciences, David Geffen School of Medicine, University of California Los Angeles, Los Angeles, CA, USA

^2^ Department of Bioengineering, University of California Los Angeles, Los Angeles, CA, USA

^3^ US MR R&D Collaborations, Siemens Medical Solutions USA, Inc., Salt Lake City, UT, USA

^4^ Physics and Biology in Medicine Interdepartmental Program, David Geffen School of Medicine, University of California Los Angeles, Los Angeles, CA, USA

^5^ US MR R&D Collaborations, Siemens Medical Solutions USA, Inc., Austin, TX, USA

^6^ Department of Pediatrics, David Geffen School of Medicine, University of California Los Angeles, Los Angeles, CA, USA

**Corresponding author:**

Holden H. Wu, Ph.D.

[HoldenWu@mednet.ucla.edu](mailto:HoldenWu@mednet.ucla.edu)

+1-310-267-6843

Department of Radiological Sciences, David Geffen School of Medicine,

University of California Los Angeles, Los Angeles, CA, USA

**Supplementary Information Table S1.** Representative imaging parameters for breath-held (BH) T_2_-weighted (T2w) Half-Fourier Single-shot Turbo spin-Echo (HASTE) and BH Volumetric Interpolated Breath-hold Examination (VIBE) Dixon. All scans were acquired in the axial orientation at 3T. TE: echo time. TR: repetition time. BW: readout bandwidth. PAT: parallel imaging. GRAPPA: Generalized Auto-calibrating Partial Parallel Acquisition, CAPIRINHA: Controlled Aliasing in Parallel Imaging Results in Higher Acceleration. N/A: not applicable.

|  | **BH T2w HASTE** | **BH VIBE Dixon** |
| --- | --- | --- |
| **TE** | 73 ms | 1.23 ms, 2.46 ms, 3.69 ms, 4.92 ms, 6.15 ms, 7.38 ms |
| **TR** | 1100 ms | 8.85 ms |
| **Flip Angle** | 150 degrees | 5 degrees |
| **Bandwidth (BW)** | 601 Hz/Px | 1170 Hz/Px |
| **Field of View (FOV)** | 380 x 310 mm^2^ | 360 x 360 mm^2^ |
| **Acquired Matrix Size** | 320 x 169 | 224 x 224 |
| **Recon. Matrix Size** | 320 x 208 | 224 x 224 |
| **Recon. Pixel Size** | 1.2 x 1.2 mm^2^ | 1.6 x 1.6 mm^2^ |
| **Interpolation** | OFF | OFF |
| **Partial Fourier** | 4/8 | OFF |
| **PAT Factor** | GRAPPA, 2 | CAIPIRINHA, 2 |
| **Slice Thickness** | 5 mm | 5 mm |
| **Number of Slices** | 47 | 26 |
| **Scan time per BH** | 13.5 seconds | 18 seconds |
| **Total Scan Time** | 53 seconds (4 BH) | 18 seconds (1 BH) |


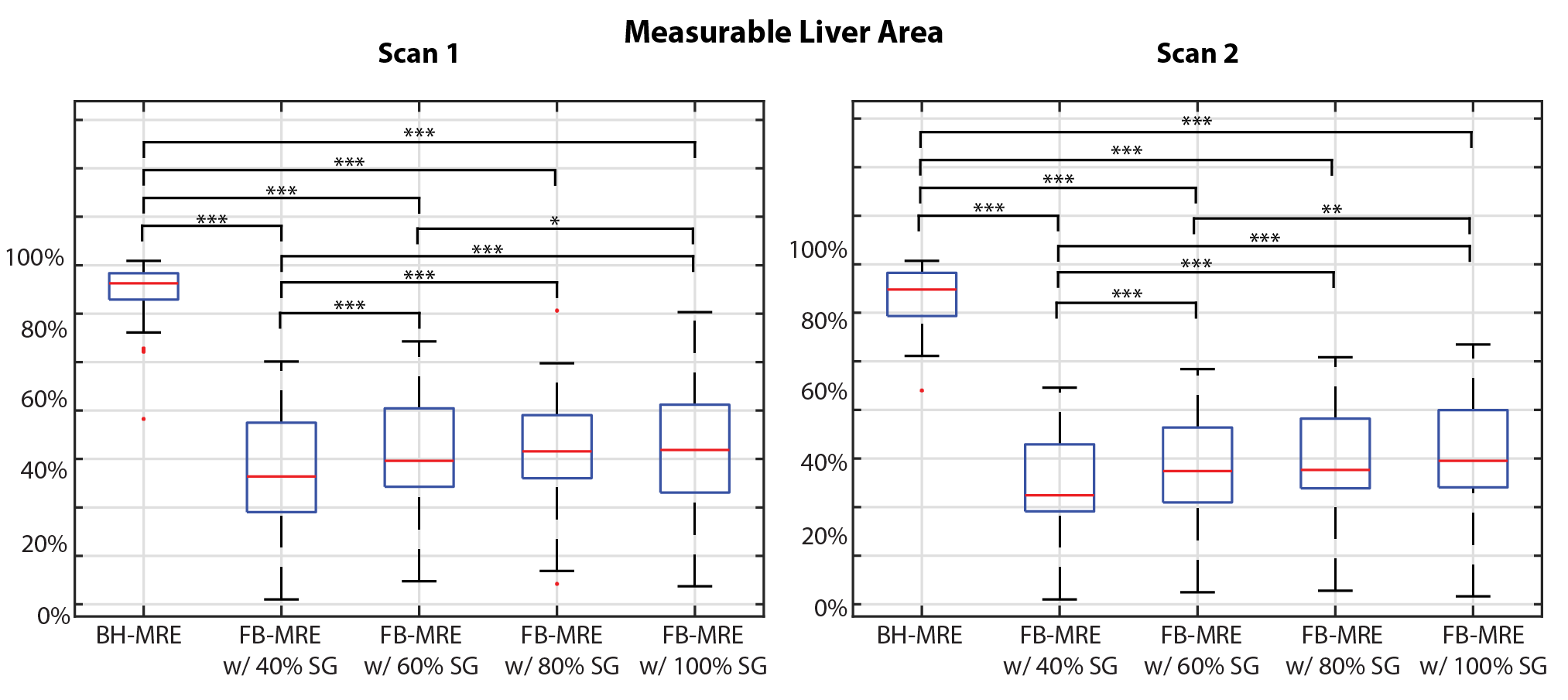


**Supplementary Information Figure S1.** Box-whisker plots of the measurable liver area on liver stiffness maps for breath-held (BH) and free-breathing (FB) MR elastography (MRE) with varying self-gating (SG) acceptance rates in scan 1 (left) and 2 (right). Pairwise comparisons of the five MRE methods yielded significant differences in all pairs except for the pairs from FB-MRE with 60% SG versus 80% SG acceptance rate and 80% SG versus 100% SG acceptance rate. * indicates $p<0.05,$ ** indicates $p<0.01$, and *** indicates $p<0.001$.
